# sRNAflow: A Tool for the Analysis of Small RNA-Seq Data

**DOI:** 10.3390/ncrna10010006

**Published:** 2024-01-17

**Authors:** Pawel Zayakin

**Affiliations:** 1 Latvian Biomedical Research and Study Centre, LV-1067 Riga, Latvia; pawel@biomed.lu.lv; 2 European Bioinformatics Institute, EMBL-EBI, Hinxton CB10 1SD, UK

**Keywords:** bioinformatics, small RNA, microbiome, non-coding RNA, biofluids, miRNA, isomiR, tRF, biomarker, cancer biology

## Abstract

The analysis of small RNA sequencing data across a range of biofluids is a significant research area, given the diversity of RNA types that hold potential diagnostic, prognostic, and predictive value. The intricate task of segregating the complex mixture of small RNAs from both human and other species, including bacteria, fungi, and viruses, poses one of the most formidable challenges in the analysis of small RNA sequencing data, currently lacking satisfactory solutions. This study introduces sRNAflow, a user-friendly bioinformatic tool with a web interface designed for the analysis of small RNAs obtained from biological fluids. Tailored to the unique requirements of such samples, the proposed pipeline addresses various challenges, including filtering potential RNAs from reagents and environment, classifying small RNA types, managing small RNA annotation overlap, conducting differential expression assays, analysing isomiRs, and presenting an approach to identify the sources of small RNAs within samples. sRNAflow also encompasses an alternative alignment-free analysis of RNA-seq data, featuring clustering and initial RNA source identification using BLAST. This comprehensive approach facilitates meaningful comparisons of results between different analytical methods.

## 1. Introduction

Next-generation sequencing (NGS) has brought about a transformative impact on various fields of biology, particularly in the realm of small RNA (sRNA) research. Small RNAs, typically less than 200 nucleotides in length, predominantly consist of non-coding RNAs engaged in cellular regulatory mechanisms [1,2,3]. Many sRNAs are even shorter, such as microRNAs (miRNAs) with a characteristic length of approximately 22 nucleotides [4] and PIWI-interacting RNAs (piRNAs) spanning 24–30 nucleotides [5]. These sRNAs exhibit altered expression profiles in different disease states, rendering them potential non-invasive biomarkers for diagnosing and monitoring various conditions, including cancer [6,7,8,9]. Circulating sRNAs have been detected in a variety of biofluids, including blood serum, plasma, saliva, urine, and cerebrospinal fluid [10].

One of the most intricate challenges in analysing RNA-seq data obtained from diverse biofluids lies in the unpredictable mixture of sRNAs originating from both human and non-human sources [11,12]. The accurate analysis of sRNA reads of human origin necessitates the separation of reads originating from other species, such as bacteria, fungi, and viruses. At the same time, the determination of the origin of small RNAs is difficult with popular utilities based on K-mers due to the small length of reads.

Traditional approaches involve mapping reads separately to the human genome or miRBase, followed by mapping the unmapped portion to microbial genomes (or vice versa). However, this two-step process poses challenges in correctly identifying the source species of sRNA reads, especially when they do not align with the highest similarity in the second alignment step after reaching a threshold in the first step. An alternative solution is a one-time alignment to a metagenome that encompasses all reference genomes.

**Figure 1:**
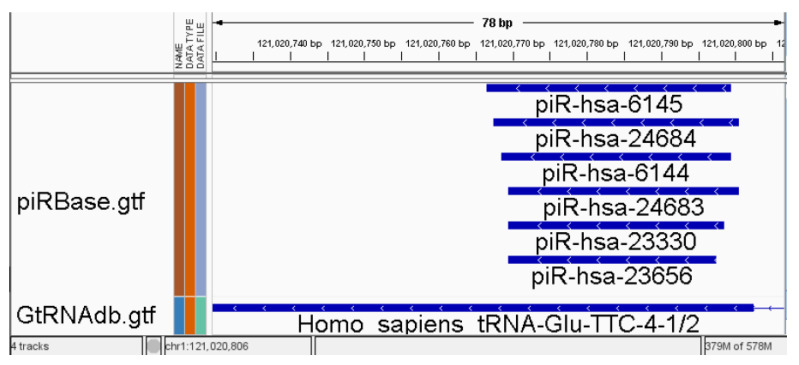
Example of overlapping annotations, demonstrating six features in piRBase [17] overlapping with a feature in GtRNAdb [20] shown in IGV [21]. All reads that are mapping to regions containing such features are identified and labelled as ambiguous by the counter.

Small RNA analysis encounters specific challenges due to overlapping features in available annotation databases [13,14,15]. This overlap conundrum forces most common counters to make a binary choice for reads mapped to such regions: they should be either marked as ambiguous and excluded from subsequent analysis or counted for all overlapping features [14,16]. This problem is particularly pronounced in uncurated databases like piRBase [17] but remains significant even in well-curated and popular databases. The issue of overlapping features, as illustrated in Figure 1, can result in cases where up to 50% of reads are marked as ambiguous (unpublished data) and are consequently excluded from the analysis. This problem can be categorised into two types: overlapping between annotations from different databases (sRNA types) and overlapping within annotations from the same source. While the second type problem is less intricate for miRNA than other RNA types, the miRTop/mirGFF3 project introduces a novel GFF3 format for the output of small RNA pipelines. This format is specifically designed to support the description of isomiRs, enabling tasks such as differential expression analysis at the isomiR level [18]. Expanding the novel annotation format to encompass other RNA types and curating databases within the RNAcentral [19] resources provided by a collaborating group of Expert Databases can be a viable solution to achieve a clearer consensus in some databases. However, it has been reported that RNAcentral does not check for overlapping piRNAs, lncRNAs, and several other ncRNA types [19].

In small RNA analysis, a substantial portion of reads is often mapped outside annotated expressed regions. Classical methods are not tailored for analysing unannotated expressed regions. Despite employing repositioning algorithms [22], up to 80% of mapped reads in certain analysed datasets [23] remained unannotated. Consequently, exploring these unannotated data can offer complementary insights to traditional biomarkers or refine biological signatures in machine learning by incorporating unknown regions. Additionally, the examined samples may contain RNA fragments whose source is from species whose genomes are not yet represented in the databases. Substantial challenges also emerge when short RNA sequences undergo post-transcriptional changes, making precise mapping difficult. The recently introduced alignment-free profiling strategy offers a solution to these challenges by bypassing the need to map reads to a reference genome [24]. Instead, actual read sequences are used to determine expression intensity. Following the differential expression analysis of individual sequences, significant sequences are annotated against user-defined databases. This strategy provides a more comprehensive representation of small RNA populations without any data loss or distortion.

The field of small RNA data analysis is rapidly advancing, with new tools for analysing specific subsets of data being published each year [25,26,27,28,29,30,31]. However, many of these tools demand computational or basic programming expertise from users.

In response to these challenges, we introduce the sRNAflow tool, which offers potential solutions to these issues. The source code can be accessed at https://github.com/zajakin/sRNAflow (accessed on 29 December 2023) under the GPL3 licence.

## 2. Materials and Methods

**Figure 2:**
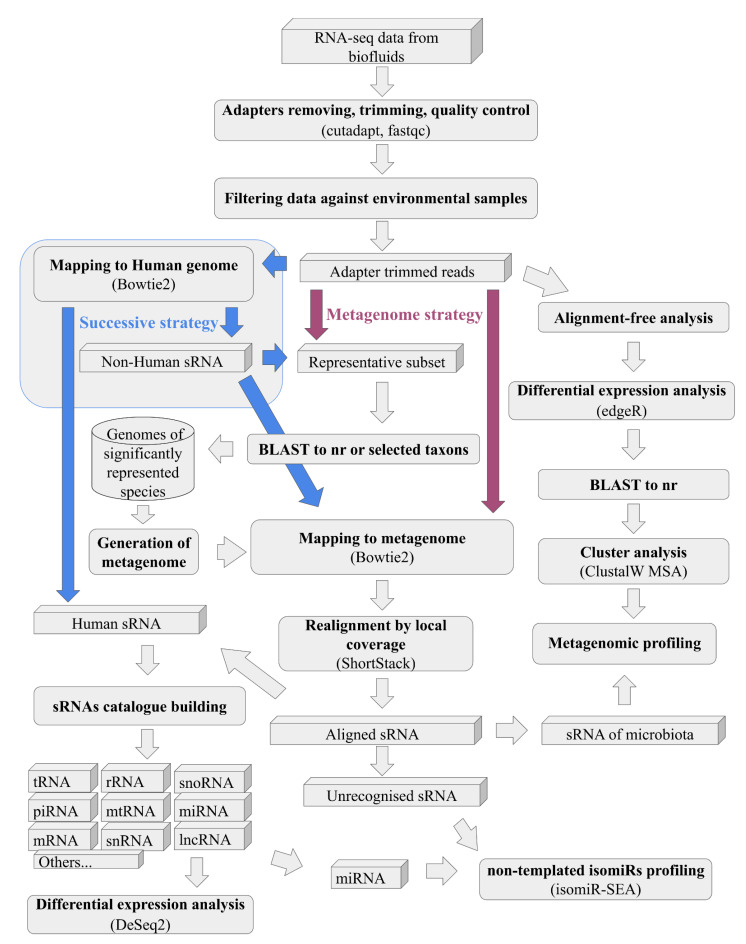
The workflow of the pipeline, with colour-coded elements highlighting the distinctions between the “Metagenome” and “Successive” strategies.

sRNAflow accepts input data in various formats, including flat or gzipped (.gz) FASTQ, FASTA, SAM, CRAM, or BAM files. The sRNAflow protocol comprises several essential steps and seamlessly integrates recognised tools into the pipeline (Figure 2):

- Adapter removal and quality trimming (cutadapt [32]);
- Quality assessment (fastqc [33]/multiQC [34]);
- BLAST of a representative subset of reads (BLAST [35]);
- Reads mapping (Bowtie [36]/Bowtie2 [37]);
- Realignment by local coverage (ShortStack [22]);
- Reads counting (Rsubread [38]);
- Differential expression analysis (DESeq2 [39]/edgeR [40]);
- Non-template isomiRs identification (isomiR-SEA [41]);
- Cluster analysis (ClustalW MSA [42]);
- Data visualisation (Krona [43]).

Specific algorithms necessary for analyses of small RNA samples from biofluids described below are included as well.

### 2.1. Installation

To install sRNAflow on a server or workstation with Docker service installed, execute the following command in your terminal:

*mkdir -m 777 sRNAflow*
*docker pull ghcr.io/zajakin/srnaflow*
*docker run -d -p 3838:3838 -v `pwd`/sRNAflow:/srv/shiny-server/www ghcr.io/zajakin/srnaflow*

After running, access the user interface in a web browser at http://<your server name or IP>:3838 (or another port if modified).

All uploads, databases, and analysis results are stored in the “sRNAflow” subfolder of the terminal’s current working folder.

### 2.2. Shiny-Based User Interface

sRNAflow is specifically designed to be user-friendly, catering to inexperienced users in the field. All necessary operations, including data upload, analysis configuration, and reports download, can be performed via the graphical user interface.

Considering that users’ desktop computers may lack sufficient resources and to enable the analysis to be run on a server, the program must have the capability to be controlled remotely. The most intuitive user interface can be facilitated via a web server.

**Figure 3:**
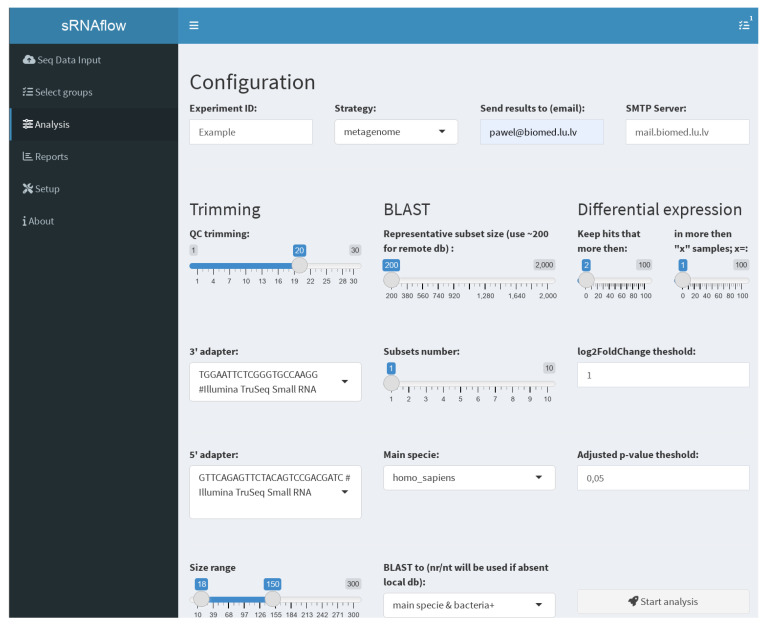
sRNAflow user interface. Analysis settings tab.

The user interface (Figure 3), built on the Shiny R package [44], facilitates file uploading, selection for analysis, grouping for differential expression analysis, local BLAST database creation, annotation file regeneration (with a primary set pre-uploaded due to its time-consuming nature), settings adjustment, and report downloading.

### 2.3. Annotations Files

Human genome annotations used in the pipeline include those from the Ensembl database, categorised by small RNA types. Additionally, annotations are sourced from miRBase [45], LNCipedia [46], piRBase [17], piRNAdb [47], GtRNAdb [20], and RepeatMasker [48].

sRNAflow provides ready-to-use annotation files and offers the option to recreate them on demand.

#### 2.3.1. Generation of Annotation Files

To recreate annotation files, access the tab “Setup” > “Update GTF files”. This operation can be time-consuming. Some databases are provided in FASTA format, and it is necessary to convert them to GTF format. To accomplish this, specific algorithms have been developed. This approach is based on 100% identity alignment without gaps to the genome, followed by SAM to GTF conversion. Moreover, a script is created to derive transfer RNA fragments (tRFs) annotation from GtRNAdb databases using tRNAscan-SE [49] output. This comprehensive annotation process ensures a diverse and detailed representation of small RNA types in the analysis.

#### 2.3.2. Merging of Overlapped Annotations Features

The challenge of overlapping features can be classified into two categories: overlap between annotations sourced from different databases (representing various sRNA types) and overlap within annotations originating from the same source. To address the first problem, a prioritisation procedure is employed. This is crucial because different types of small RNAs are explored to varying degrees, and uncurated databases for some of them likely contain erroneous entries. The prioritisation algorithm used to construct a catalogue of expressed RNA types resolves issues arising from the use of different annotation databases, particularly addressing problems related to overlapping annotations. The prioritisation algorithm, integrated into the pipeline’s default settings, follows the priority order adopted from [31] and has been updated for additional RNA type annotations: miRNA > tRNA > rRNA > mRNA > processed pseudogenes > snRNA > snoRNA > mtRNA > piRNA > lncRNA > vaultRNA > Y RNA > other types RNA > repeats and low complexity RNA (tRNA > rRNA > other).

The second problem of overlapping features within the same annotations file is resolved by merging them into a unified feature, with corresponding changes to its name and attributes.

### 2.4. Using the Pipeline

sRNAflow itself does not consume a significant amount of memory or CPU resources, but the BLAST alignment, especially with a local database, can be CPU and time-intensive. The creation of the Bowtie2 index for a large metagenome database can also be resource-intensive. It is advised to create a local BLAST database using the button in the “Setup” tab to enable filtering by taxa, an option not available with remote databases, and to reduce the likelihood of selecting incorrect species. We recommend using a system with at least 30 GB of memory. To regulate CPU usage, include the flag “--cpus x” in the command, where x denotes the number of allowed cores.

To use the pipeline, follow these steps:

#### 2.4.1. Data Upload

Upload your data in flat or gzipped (.gz) FASTQ, FASTA, SAM, CRAM, or BAM files on the “Seq Data Input” tab. Alternatively, select files already situated on the server or use Example files. Click on uploaded files to select for analysis or remove the selection.

#### 2.4.2. Group Selection

On the tab “Select groups”, choose a group (test, control, environment, or ignore) for each selected file.

#### 2.4.3. Analysis Options

On the tab “Analysis” (Figure 3), you can set the necessary options:

- Trimming—used adapters, size, and quality (QC) limits;
- BLAST—Switch taxa filter option for local database and number and size of the representative subsets. This selection is a tradeoff between resource consumption and the sensitivity of the pipeline to detect a rarely represented species in the sample. We recommend starting with a size of 200 reads, especially for a remote BLAST database and increasing if necessary.
- Differential expression—thresholds to filter expressible RNA (sequence in alignment-free analysis) and log2FoldChange and adjusted *p*-values to filter out statistically insignificant results.
- Strategy of the pipeline (Figure 2), where, in the case of “metagenome”, all reads at once will be mapped to generated on BLAST results metagenome or more traditional “successive” strategy, where, at first, samples mapped to the human genome and only reads unmapped to it will be mapped to the generated metagenome.
- Provide an email address and mail server if you prefer to receive notifications and report files on email.

#### 2.4.4. Analysis Start

Start analysis and check for report files that are accessible in the “Reports” tab.

### 2.5. Filtering of Environmental Contamination

Filtering data against environmental samples is an essential step that has a profound impact on the results obtained. These environmental samples, excluding biological material, encompass all components used in RNA extraction and library preparation specific to the current laboratory. This ensures data accuracy and reliability and should be planned during experiment design. In our pipeline, we exclude all reads that have sequences identical to those from environmental samples or include those sequences as part of their composition. This rigorous filtering approach enhances the precision of our analysis by minimising the influence of potential contaminants.

### 2.6. Source of Presented Small RNA Recognition

Users should choose one of two strategies: the newly proposed “metagenome strategy” algorithm designed to minimise the false-positive matching of reads to improper species, where reads are mapped to host species and microbiomes in one turn, and a more traditional “successive strategy”, where, at first, samples mapped to the human genome and only unmapped to its reads will be mapped to the generated metagenome. Analysis initiates with a two-pass analysis based on the BLAST [35] output on the entire “nr” database or specified taxons (only available for locally downloaded BLAST databases, accessed through “Setup” > “Create/Update local BLAST DB”). This analysis employs a representative random subset of reads, and the size of this subset should be determined by the user during the analysis setup step. Caution is advised, particularly for remote databases with a subset size larger than 200 reads. This choice involves a tradeoff between resource consumption and the pipeline’s sensitivity to detect species that are infrequently represented in the sample. While this approach may inherently introduce some false-positive results, refinement is possible by adjusting the list of comparable taxa. The current list encompasses host species and taxonomic trees of potential host parasites and microbiomes (The taxa IDs is given in brackets):

- Homo (9606);
- Bacteria (2);
- Fungi (4751);
- Viruses (10239);
- Archaea (2157);
- Amoebozoa (554915);
- Discoba (2611352);
- CRuMs (2608240);
- Metamonada (2611341);
- Sar (2698737);
- Eukaryota incertae sedis (2683617);
- Aphelida (2316435);
- Ichthyosporea (127916);
- Rotosphaerida (2686024);
- other sequences (28384).

Identified species are ranked by their frequency of presence in the sample. In the second pass, the hit is assigned to the species with a higher rank in the first pass in case of a similar obtained BLAST score. Simultaneously, valuable research information on accompanying species is obtained and visualised using Krona 2.8.1 [43].

### 2.7. Metagenome Generation and Alignment

Only the genomes of the most represented species in successful BLAST hits, covering more than 1% of the hits, will be utilised for subsequent alignment steps. Genomes of these reported species are downloaded in FASTA format from RefSeq, Ensembl, or GenBank and added as additional entries with accordingly changed descriptions. The size of the constructed metagenome can vary based on the number of genomes used and may reach 200–300 Gb in some cases. In such instances, the generation of mapper indices will be memory-consuming and require a substantial memory server.

Unlike full-length mRNA, sRNA reads typically align in multiple sites of the genome. Our pipeline aligns the reads using Bowtie 2 [37], allowing for multiple alignments per read. Subsequently, the reads are reassigned, taking into account local coverage, using the ShortStack algorithm [22].

### 2.8. Small RNA Types and Identified Species Catalogues

This pipeline includes the creation of a catalogue of expressed RNA types, utilising human genome annotations as a percentage of all non-intronic identified features. This catalogue is a valuable resource for understanding and characterising the diverse landscape of expressed RNA types in the analysis.

A catalogue of identified species was prepared, presenting assigned read counts for detected species in all samples. The visualisation of identified species for each sample was performed using Krona 2.8.1.

### 2.9. Differential Expression Analysis

Differential expression analysis was performed by DESeq2 [39] for classifiable RNA types (miRNA, piRNA, tRNA, and other sRNAs) when analysing two sample groups.

### 2.10. Alignment-Free Sequence Analysis

In our proposed pipeline, we adopted an approach that includes alignment-free analysis of RNA-seq data, featuring clustering and the initial identification of the RNA source, similar to the R DEUS package [24], with variations in additional data filtering. Notably, the adjustable “keep hits” variable enables a substantial reduction in the analysed dataset, leading to accelerated creation and analysis times. In the subsequent step, we focus on the most significantly changed sequences, limiting the analysis to no more than 1000 upregulated and 1000 downregulated sequences. These sequences are then clustered using ClustalW 2.1, and initial RNA source identification using BLAST 2.5.0 is performed. The ultimate goal is to obtain consensus sequences that unite clustered sequences. The final selection at this stage is carried out manually.

### 2.11. Reports

- A consolidated Excel file report is presented, encompassing a comprehensive set of information (example report attached as Supplementary File S1):
  ○ Analysis settings;
  ○ Sample and trimming statistics;
  ○ A catalogue of identified species;
  ○ A catalogue of sRNA types;
  ○ Counts of identified features;
  ○ Spearman sample correlation tables with heatmap visualisation;
  ○ Differential expression analysis for annotated RNA types;
  ○ The file includes visualisations such as Volcano [50] and PCA plots.
- Quality Diagrams:
  ○ Sample quality diagrams, generated by fastQC [33] and consolidated by multiQC [34], are provided as downloadable zip files.
- Alignment-free Analysis (example report attached as Supplementary File S2):
  ○ Results of the alignment-free analysis of RNA-seq data, featuring clustering and the initial identification of the RNA source, are presented in a separate Excel file.
- Post-translational Modifications and Enrichment Analysis, formatted in Excel for user convenience (example report attached as Supplementary File S3):
  ○ Acknowledging the significant role of miRNA and other sRNA post-translational modifications in adaptive regulation [51,52], the pipeline includes the identification of non-template isomiRs using isomiR-SEA [41].

## 3. Results and Discussion

The identification of differentially expressed small RNAs holds considerable diagnostic potential, particularly in cancer and cardiovascular diseases [6,7,8,9,53]. A preliminary iteration of the presented program has been utilised in recent publications focusing on the analysis of extracellular vesicle contents, engaging in discussions about the biological interpretations of the results [51,53,54].

### 3.1. Merging of Overlapped Annotations Features

Versions of databases of small RNA used in the current pipeline and the results of merging overlapped annotation features are detailed in Table 1. The last column indicates the percentage of features merged per database. This step reveals a significant divergence in the necessity of this operation for various RNA types, ranging from being completely unnecessary for tRNA to being highly essential for mRNA and lncRNA exons, which, as fragments, can be present in small RNA samples. Notably, the proposed approach successfully addresses challenges posed by overlapped annotation features, ensuring that reads and features excluded in conventional methods as ambiguous are retained in the analysed samples. This underscores the tool’s ability to preserve valuable information in complex datasets.

The latest version of piRBase (version 3, [55]) requires additional preparation to be integrated into the described pipeline. Its rapid size growth underscores the necessity to transition from individual piRNA analysis to piRNA cluster analysis [56]. This shift is motivated by findings demonstrating contamination of many piRNA databases with non-coding RNA fragments, particularly in somatic tissues. Notably, the piRNA cluster database stands out for its remarkably low incidence of such contaminations [57]. We are assessing a transition to the analysis of piRNA clusters in future versions of the pipeline.

### 3.2. Testing the BLAST-Based Approach on Simulated Positive and Negative Controls

Since experimental samples cannot be guaranteed to be free of any contamination, as a positive and negative control of the method, we simulated samples from the Escherichia coli and Homo sapiens ncRNA databases using the R package polyester [58] with a length variation of 10–44 bp. These samples are provided with the Docker image. sRNAflow showed for both of them 99% sensitivity and 99% specificity for a sample simulated from E. coli data where 1% of reads with lengths of 15–17 bp were identified as human. In the sample simulated from human data, 0.7% of reads with lengths of 16–23 bp were not identified.

### 3.3. Example sRNAflow Reports on a Simulated Dataset

The efficiency of the sRNAflow tool has been tested using a simulated short read dataset (10 samples with 10,000 reads in each) was established based on the experimental samples from the sequencing of sRNAs in plasma and urinary extracellular vesicles from a longitudinal cohort of 20 prostate cancer patients [54]. Inside the user interface, as an additional example, RNA-seq data obtained by sequencing small RNAs from urinary cells and extracellular vesicles can be downloaded [52].

**Figure 4:**
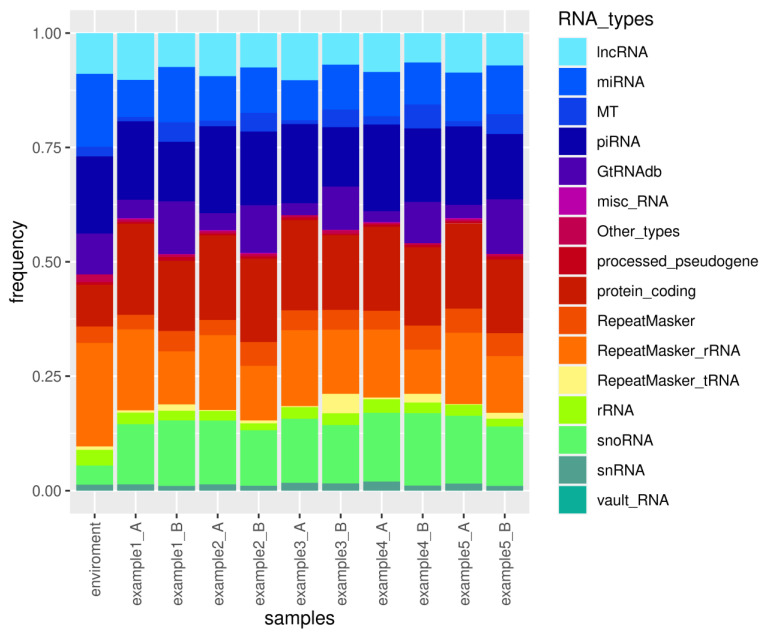
Catalogue visualisation of small RNA types annotated in the simulated dataset.

The consolidated Excel file report, generated by sRNAflow and attached as Supplementary File S1, includes sample and trimming statistics, a catalogue of identified species, a catalogue of sRNA types (Figure 4), counts of identified features, Spearman sample correlation tables with heatmap visualisation, and differential expression analysis performed by DeSeq2 for annotated RNA types. The visual representation of the catalogue of small RNA for the simulated dataset (Figure 4) illustrates the variability in the proportion of different small RNA types. The results presented in the report indicate statistically significant differential expression for only one miRNA, one mRNA, three snoRNAs, four piRNAs, and three lncRNAs. This limited number of differentially expressed RNAs can be attributed to the small size of the simulated samples.

Supplementary File S2 provides an Excel file with alignment-free reports showcasing the results of the alignment-free differential expression analysis of small RNA-seq data. The file includes visualisations such as Volcano and PCA plots, along with clustering performed by ClustalW and the initial identification of the RNA source using BLAST. While only 12 RNAs annotated as statistically significant differentially expressed are presented above, the alignment-free analysis reveals 73 statistically significant differentially expressed sequences, with 30 of them preliminarily identified as human and 24 as bacterial sources. Some of these may represent isoforms of small RNA, as alignment-free analysis compares expression levels of completely identical sequences.

Supplementary File S3 contains results of post-translational modifications and enrichment analyses generated by isomiR-SEA [41], specifically focusing on the identification of non-template isomiRs.

### 3.4. Comparison of Small RNA Analysis Pipelines on a Simulated Dataset

Using a simulated dataset, we conducted a performance comparison of sRNAflow with several programs designed for small RNA analysis and traditionally used pipelines. These include sMETASeq [30], exceRpt [31], and sRNAtoolbox [29], which is well-known and encompasses analysis of microbiome and host small RNAs. Kraken2 is a taxonomic classification system using exact k-mer matches, widely used in the analysis of microbiomes. The pipelines that include cutadapt, bowtie2, and Rsubread are also widely recognised for sRNA analysis of human RNA-Seq data [59,60].

The results of sMETASeq [30], as presented in Table 2, show a significant alteration in the proportion of ambiguous reads depending on the source of annotations utilised. This effect is attributed to the overlapping of annotations of different RNA types included in the RNACentral annotation file supplied with sMETASeq, whereas miRBase includes only miRNA annotations. The results, shown in the “Annotated human” column, highlight the significant efficiency of the RNA type prioritisation procedure employed by sRNAtoolbox [29], exceRpt [31] and sRNAflow, which yields 30–46% of annotated reads compared to 1–15% when such a procedure is not employed. Significantly, sRNAflow exhibits a slight improvement over sRNAtoolbox, which follows closely at 42%, in this metric, showcasing a performance of 44–46%. The results demonstrate a distinction between the two strategies employed in sRNAflow, specifically in the identification of certain reads as either human or microbiome. In the successive strategy, these reads are identified as human reads, moreover, in annotated areas. In the metagenome strategy, aligning in a one-time alignment to a metagenome that encompasses all identified reference genomes, these same reads align with the highest similarity to the microbiome. This difference undoubtedly influences the selection process during the subsequent stages of the differential expression analysis.

The most effective outcomes in the comprehensive identification of the source of reads, with the minimum value observed in the “Unidentified” column (13%), are achieved using sRNAtoolbox. Subsequently, sRNAflow follows closely with a percentage of 19%.

The demonstration of the presence of all types of RNA in the environmental sample, as illustrated in Figure 4, along with the proportion of reads filtered out (Table 2) due to their close similarity to reads in the environmental sample, underscores the importance of incorporating such samples into experimental design when isolating small RNAs. Contamination sources may vary for each laboratory, set of reagents, or operator. Therefore, we suggest that the preparation of environmental samples should be included in the experimental design, encompassing all components used in RNA extraction and library preparation.

**Figure 5:**
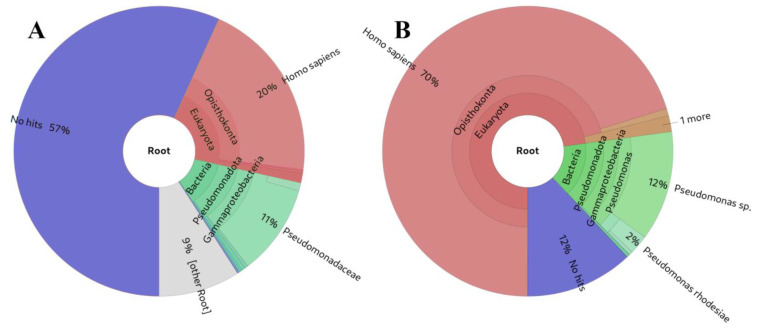
Comparison of small RNA source identifications from the same example from the dataset of small RNA-Seq simulated based on experimental data: (**A**) Kraken2 [61], (**B**) sRNAflow approach based on BLAST.

The analysis of the source of the presented small RNA results in Table 2 suggests that, overall, our approach and other alignment-based methods exhibit greater sensitivity (Figure 5B) for small RNA analysis compared to Kraken2 (Figure 5A) [61] or MetaPhlAn [62] (not shown in Table 2, as it did not detect any taxa in the simulated samples). This heightened sensitivity is attributed to the fact that the K-mers used to construct their databases must be longer (35-mers, 100-mers, 150-mers, and 200-mers) than the majority of the small RNA-derived reads. This requirement is essential to prevent program usage from becoming impractical due to high resource utilisation. While some false positive hits are observed, particularly for the shortest small RNAs, this outcome was anticipated as these sequences can be identical across various biological species.

### 3.5. Analysis of Microbiome in Ancient DNA samples

**Figure 6:**
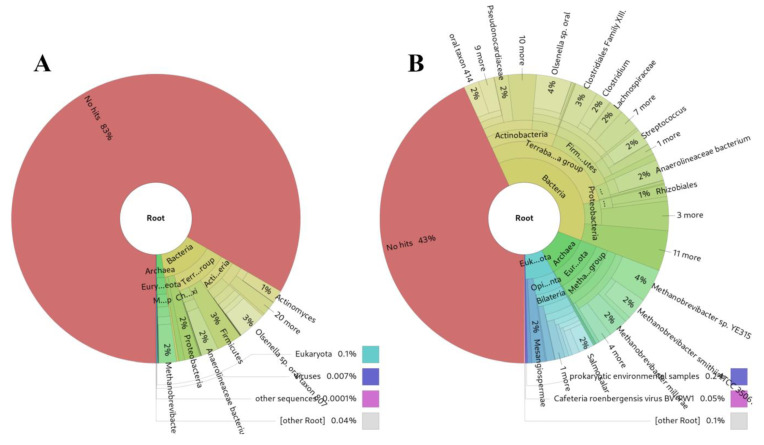
Comparison of source identifications for archaeological DNA from the same microbiome DNA shotgun samples dating back to the XVI-XVII centuries [63]: (**A**) Kraken2, (**B**) sRNAflow approach based on BLAST.

An unexpectedly successful outcome of sRNAflow was its application to 20 archaeological microbiome DNA shotgun samples dating back to the XVI-XVII centuries [63,64]. Our approach demonstrates more sensitive results (Figure 6B) compared to Kraken2 [61] (Figure 6A) for highly damaged DNA, presumably due to substantial degradation over time.

## 4. Conclusions

The presented pipeline for small RNA analysis is a user-friendly bioinformatic tool with a graphical user interface designed for non-programmer users. Tailored for the specific demands of small RNA analysis, this pipeline addresses various challenges. It includes features for filtering potential contaminant RNAs from the environment, categorising small RNA types, handling overlap in small RNA annotations, conducting differential expression assays, analysing isomiRs, and an approach to identify the sources of small RNAs within samples. Additionally, it offers an alternative alignment-free analysis of RNA-seq data, incorporating clustering and initial RNA source identification using BLAST.

## Figures and Tables

**Table 1:** Human genome annotations used in the pipeline: results of merging overlapped annotation features. the Ensembl database is categorised by small RNA types.

Database	Version	Accessed	Format	Features	Merged	% Merged
miRBase_hairpin	v22	December 2013	GFF3	1918	1859	3
miRBase_mature		December 2013	GFF3	2883	2813	2
GtRNAdb	v21	December 2013	FASTA	432	432	0
LNCipedia	v5.2	December 2013	GTF	357,620	151,562	58
LNCipedia_hc		December 2013	GTF	288,174	127,290	56
piRNAdb	v1.7.6	December 2013	FASTA	814,994	558,329	31
piRBase	v1	December 2013	FASTA	797,231	549,328	31
Ensembl	GRCh38.p14	December 2013	GTF	1,649,690	345,110	79
miRNA				1879	1822	3
rRNA				53	53	0
protein_coding				1,387,673	235,196	83
processed_pseudogene				11,773	11,731	0
snRNA				1910	1910	0
snoRNA				942	925	2
MT				37	32	14
lncRNA				217,724	71,419	67
vault_RNA				1	1	0
YRNA				814	814	0
notY_misc_RNA				1407	1407	0
Other_types				25,477	19,800	22
RepeatMasker	Gencode v44	December 2013	FASTA	5,683,690	5,536,563	3
RepeatMasker_tRNA				2164	2164	0
RepeatMasker_rRNA				565	538	5

**Table 2:** Comparison of proportions of identified and annotated reads in simulated dataset by different small RNA analysing pipelines.

Pipeline	Filtered QC and <15 bp	Filtered Environment	Annotated Human	Ambiguous Human	Unannotated Human	Identified Other Species	Unidentified
sMETASeq (RNACentral)	14%	-	1%	17%	41%	5%	22%
sMETASeq (MiRBase)	14%	-	6%	0.01%	52%	5%	22%
Cutadapt + Kraken2	14%	-	-	-	13%	13%	59%
Cutadapt + bowtie2 + Rsubread(Ens.) + Kraken2	14%	-	15%	12%	25%	6%	26%
exceRpt	36%	-	30%		4%	-	30%
sRNAtoolbox	14%	-	42%		14%	17%	9 * + 4%
sRNAflow (metagenome)	14%	-	44%	0%	5%	16%	19%
sRNAflow (successively)	14%	-	46%	0%	6%	14%	19%
Pipelines that include filtering against an environmental sample							
Cutadapt + Kraken2	14%	28%	-	-	7%	11%	40%
Cutadapt + bowtie2 + Rsubread(Ens.) + Kraken2	14%	28%	7%	5%	15%	6%	25%
sRNAflow (metagenome)	14%	28%	19%	0%	6%	15%	18%
sRNAflow (successively)	14%	28%	21%	0%	6%	13%	18%

* The status of these reads is not clear from the files provided by sRNAtoolbox.

## Data Availability

Project data and Source Code available under GPL3 license: https://github.com/zajakin/sRNAflow (accessed on 29 December 2023).

## Supplementary Materials

The following supporting information can be downloaded at https://www.mdpi.com/article/10.3390/ncrna10010006/s1. File S1: Example of consolidated Excel file report; File S2: Example of Alignment-free Analysis report; File S3: Example of Post-translational Modifications Analysis report.
